# Lignin Depolymerization under Continuous‐Flow Conditions: Highlights of Recent Developments

**DOI:** 10.1002/cssc.202001225

**Published:** 2020-07-09

**Authors:** Omar Y. Abdelaziz, Christian P. Hulteberg

**Affiliations:** ^1^ Department of Chemical Engineering Lund University Naturvetarvägen 14 221 00 Lund Sweden

**Keywords:** continuous processing, depolymerization, flow chemistry, hydrothermal conversion, lignin valorization

## Abstract

Lignin is a polyaromatic polymer contained in plant cell walls, and it is considered the most abundant noncarbohydrate polymer on Earth. The aromaticity and richness of its functional groups render lignin an attractive starting biomacromolecule for conversion into a variety of value‐added products. The development of successful strategies for lignin valorization infers the design of effective depolymerization protocols. Most research on lignin depolymerization has focused on batch‐mode processing, whereas only a few studies have investigated such lignin transformations in continuous reactor systems. In this Concept, emerging developments within the concept of continuous lignin processing and the challenges remaining in realizing the efficient valorization of lignin by using this technology are highlighted. A special focus is set on the hydrothermal conversion of technical lignin under continuous‐flow conditions, together with suggestions for future research and technology development.

## Graphical abstract

**Ready for the flow**: The latest advances in the field of hydrothermal depolymerization of technical lignin under continuous‐flow conditions are summarized and the challenges remaining in realizing the efficient valorization of lignin by using this technology are underlined.

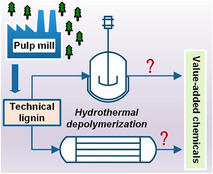

The production of renewable fuels and chemicals from biomass‐based resources is essential to drive the transition to a more sustainable chemical industry. Among different routes offered by nature to catalyze the transition, lignin, being the most abundantly available biopolymer, fosters numerous opportunities for producing valuable aromatic building blocks. Lignin is also a major industrial side product generated by pulp and paper mills and cellulosic ethanol production, presenting a bulk feedstock referred to as technical lignin.^[1]^ Developing economic pathways for the conversion of lignin into value‐added chemicals and fuels is among the largest hurdles associated with lignin valorization.^[2]^ Evolving lignin‐depolymerization approaches are providing promising opportunities for the efficient conversion of this renewable raw material into useful compounds.[^3^, ^4^] However, the step to take this fundamental research to practical applications in the heart of the biorefineries represents a major challenge.^[5]^

The aim of this Concept is to briefly highlight the latest developments in the area of continuous lignin depolymerization. The hydrothermal depolymerization of technical (industrial) lignin streams into low‐molecular‐weight aromatic compounds is particularly underlined. A performance comparison between batch and continuous systems for lignin depolymerization is presented, pointing out the major challenges encountered in both operations. A variety of emerging studies have been reported on other continuous lignin depolymerization strategies, such as hydrogenation and lignin‐first biorefining.[^6^, ^7^] However, they are beyond the scope of this Concept.

In the last decade, very few research initiatives have tackled the development of continuous‐flow reactor systems for the hydrothermal depolymerization of technical lignin into valuable phenolic compounds (Figure 1). In the same recent timeframe, various analogous studies have been described that have employed batch‐mode operations. An overview of the studies on such lignin conversion routes in continuous mode is given in Table 1.

**Figure 1:**
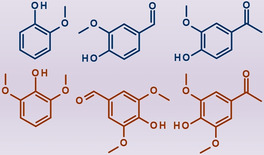
Representative one‐ring phenolic intermediates resulting from the hydrothermal fragmentation of lignin by flow‐through systems.

**Table 1:** An overview of lignin processing in flow‐through reactors.

Lignin	Concentration [wt %]	*T* [°C]	*P* [bar]	Residence time [min]	Reactor module^[a]^	Ref.
Acid hydrolysis	3	300, 350	200	20–46.7	CSTR	[14]
Kraft (LignoBoost)	12	300	180	8–24	PFR	[12]
Kraft (Indulin AT)	5	170–250	120–130	1, 2, 4	PFR	[10]
Organosolv; kraft (BioChoice)	2.5–10	250–340	250	7.5–15	PFR	[13]
Kraft (LignoBoost)	5.5	350	250	11	PFR	[11]
Kraft (Indulin AT)	10	270, 290, 315	130	15, 23, 43	PFR	[9]
Organosolv	2.5–10	240–340	250–315	2.5–15	PFR	[8]

[a] CSTR=continuous stirred‐tank reactor; PFR=plug‐flow reactor.

Roberts et al.^[8]^ reported a method for base‐catalyzed depolymerization (BCD, hydrothermal lignin treatment below the supercritical state in alkaline media) of organosolv lignin in a continuous‐reactor setup equipped with a water preheater and a cooler. Their study was the first to show that phenolic monomers are the primary products of lignin depolymerization under alkaline conditions, whereas the oligomers are the result of successive condensation reactions between these highly reactive products. This was done by analyzing the reaction products at various residence times and making conclusions based on the product formation. Beauchet et al.^[9]^ investigated the BCD of a commercial kraft lignin (Indulin AT) in a continuous‐flow reactor electrically heated by a molten eutectic potassium nitrate/nitrite salt, demonstrating that NaOH as a catalyst is efficient for the production of maximal yields of monomers that are suitable for the production of liquid chemicals. Indulin AT was also used as a feedstock for the production of low‐molecular weight aromatics through BCD under continuous‐flow conditions, as described by Abdelaziz et al.,^[10]^ notably in about 2 min reaction time and without using any organic solvent or capping agent.

Nguyen et al.^[11]^ demonstrated, in a pilot study, the conversion of LignoBoost kraft lignin into bio‐oil and chemicals in near‐critical water, where the transformation took place in a continuous fixed‐bed catalytic reactor (500 cm^3^) filled with ZrO_2_ pellets using K_2_CO_3_ as a cocatalyst and phenol as both a cosolvent and a capping agent. The products consisted of an aqueous phase, containing phenolics, and a bio‐oil, with an increased heat value (32 MJ kg^−1^) in reference to the lignin feed. Otromke et al.^[12]^ investigated the continuous BCD of LignoBoost lignin, with the objective to optimize the conversion for the synthesis of a sustainable carbon fiber precursor. In their process, an oily liquid phase containing valuable catechol and methylated derivatives was generated with yields of roughly 10 wt %. Rößiger et al.^[13]^ investigated the upscaling of BCD of hardwood organosolv lignin and softwood kraft lignin to pilot plant dimensions. Bio‐oils rich in phenolic monomers, such as guaiacol, catechol, and/or syringol, were obtained in yields of up to 13.3 wt % and 14.5 wt % from organosolv lignin and kraft lignin, respectively.

It should be noted that the aforementioned studies were all carried out using plug‐flow reactor (PFR) modules. Recently, Kristianto et al.^[14]^ demonstrated a process for converting acid hydrolysis lignin into a low‐molecular‐weight bio‐oil using a continuous stirred‐tank reactor (CSTR). An enhanced yield of bio‐oil and a suppression of solid residue were achieved compared to batch operation and were attributed to the fast heating rate. Compared to PFRs, CSTRs can offer improved mixing and controllability. CSTRs may also be favored over PFRs in the hydrothermal conversion of lignin due to the vulnerability of tubes to plugging from carbon deposits. To our knowledge, this represents the first study on hydrothermal lignin conversion using a CSTR reactor module. Such developments in the reactor design presents a foundation for possible industrial implementation of a lignin conversion technology, with potentially viable business scenarios.

As regards the chemical structure, technical lignins are characterized by being very complex and highly heterogeneous. The inherent heterogeneity of native lignin is additionally coupled with the variability and the complex chemistries of the delignification processes used in its isolation, for example, kraft pulping.^[15]^ The structure complexity resulting depends also on the botanical origin,^[16]^ and this in many cases pose challenges on their effective transformation and valorization.

Further challenges can occur because of the rather high degree of complexity, diversity, and variability of the generated product mixtures. Such products are seldom suitable for direct utilization, and innovative separation and upgrading processes are thus required. Membrane separation of depolymerized lignin represents a promising approach for product fractionation and upgrading, by which well‐defined functional fractions could be obtained.[^17^, ^18^] Compared to other available separation techniques, such as acidification, solvent extraction, and adsorption, membrane separation has the advantage of separating compounds based on molecular weight without using excessive amounts of solvents. Another promising approach is biological lignin valorization,[^19^, ^20^] which aims at exploiting the multitude of aromatics resulting from chemical depolymerization through funneling to one or two intermediates. The strategy relies on engineered microbial platforms that can uptake and metabolize such lignin‐derived intermediates for further conversion into value‐added compounds, such as *cis*,*cis*‐muconic acid and 2,4‐ or 2,5‐pyridinedicarboxylic acid.^[21]^

Understanding and improving technical lignin transformations under continuous‐flow conditions could facilitate the transition towards industrial application. The continuous depolymerization of lignin has a number of advantages compared to batch‐mode processing (Figure 2). Continuous‐flow reactor systems are able to allow shorter processing times, thus lowering the overall energy consumption. Less than 30 min of residence time is often sufficient to enable an effective lignin depolymerization process in a continuous‐flow reactor system. This is unlike the corresponding batch processes, which usually require at least 1 h of residence time to drive the transformation. Another advantage to processing lignin in flow‐through reactors is the rapid heating and cooling rates. This is an important aspect as some of the reactions relevant to lignin depolymerization may happen during preheating or cooling times apart from the desired reaction temperature. Rapid quenching could also suppress unwanted reactions that could occur and form more complex lignin structures, that is, repolymerization. An additional gain with continuous‐flow reactors is the relatively simple possibility of heat recovery through exchanging the reactor exit stream with the stream going into the reactor, enabling conditions for extra energy saving, which is in line with the principles of green engineering and sustainable process design.

**Figure 2:**
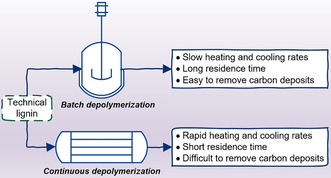
Characteristics of batch and continuous systems for lignin depolymerization.

In light of the work summarized above, the authors strongly feel that the continuous processing concept is an important stepping‐stone to technical lignin valorization. New processes with improved sustainability profiles could potentially be enabled for bio‐based chemical production on an industrial scale. Further research is needed to include optimization of the operating conditions, addressing the formation and removal of carbon deposits, reactor design for scale‐up, design of heat recovery solutions, and perhaps also process intensification solutions with the ability of cofeeding hydrogen or oxygen‐rich gas to the reactors.

## Conflict of interest

*The authors declare no conflict of interest*.
